# Architectural Design Qualities of an Adolescent Psychiatric Hospital to Benefit Patients and Staff

**DOI:** 10.1177/19375867231180907

**Published:** 2023-06-26

**Authors:** Neda Norouzi, Antonio Martinez, Zayra Rico

**Affiliations:** 1 Department of Architecture, University of Texas at San Antonio, TX, USA

**Keywords:** architecture, adolescent patients, psychiatric hospital, environmental design, evidence-based design, mental health

## Abstract

**Objectives:**

This study is focused on how architectural design of adolescent psychiatric hospitals could positively affect not only patients but also staff members working at the hospitals.

**Background:**

Adolescents between the ages of 12 and 18 are among the young population with the highest percentage of mental illness. However, there are limited number of intentionally designed psychiatric hospitals for adolescents. Staff who work in adolescent psychiatric hospitals may face workplace violence. Studies on environmental impacts suggest that the built environment affects patients’ well-being and safety as well as staff’s satisfaction, working condition, safety, and health. However, there are very few studies that focus on adolescent psychiatric hospitals and the impact of the built environment on both staff and patients.

**Methods:**

Data were collected through literature analysis and semi-structured interviews with staff of three psychiatric state hospitals with adolescent patient units. The triangulation of multiple data sources informed a set of environmental design conditions that captures the complexity and connectedness of architectural design and the occupants of an adolescent psychiatric hospital.

**Results:**

The results present architectural composition, atmosphere, lighting, natural environment, safety, and security as indispensable design conditions to create an enclosed and city-like campus that provides a serene, secure, and structured environment that benefit staff and adolescent patients.

**Conclusion:**

The specific design strategies that need to be incorporated in the architectural design of a safe and secure adolescent psychiatric hospital include an open floor plan that respects patients’ autonomy and offers privacy while always providing staff with full visibility of patients.

## Introduction

Adolescents between the ages of 12 and 18 are among the young population with the highest percentage of mental illness (McGorry et al., 2013). However, only 20% of the 15 million American adolescents with mental illness receive care with less than half of them receiving proper treatment (Jones, 2019). One of the main challenges contributing to the limited services is the inadequate design of adolescent psychiatric hospitals (McGorry et al., 2013). The impact of the built environment on patients’ mental health was first studied in the 1950s (Evans, 2003). Today, after almost a century, there is still limited information focused on the cultural and developmental needs of adolescent psychiatric patients from the built environment (McGorry et al., 2013). This study focuses on improving adolescent psychiatric hospital design in a way that directly responds to their needs.

There are major differences between the needs of adolescent and children or adults in a psychiatric hospital. Although individuals under the age of 18 are considered children and may all share the need of parental support, adolescents require more control and peer socialization (Borg-Laufs, 2013). While previous studies have proven the impact of the built environment of a psychiatric hospital on patients’ positive interactions and feeling of control and safety, there is a lack of focus on designing adolescent-specific psychiatric hospitals. This has led to many adolescents getting admitted to adult or children’s units (Blumberg & Devlin, 2006; Hutton, 2005).

In a psychiatric hospital, adolescents spend most of their time in a patient unit under strict supervision by staff (Chun et al., 2016). Although this strict supervision is necessary for patients’ safety, it often leads to aggression and severely disruptive behavior (Hallman et al., 2014) toward the staff member working at the hospital. Staff who work in adolescent psychiatric hospitals may face multiple forms of hostility, aggression, and assault, collectively referred to as workplace violence that is often triggered by patients (Hallman et al., 2014; Ulrich et al., 2018). Studies on environmental impacts suggest that the built environment affects patients’ well-being and safety as well as staff’s satisfaction, working condition, safety, and health (Sheehan et al., 2013; Trzpuc et al., 2016). However, most previously published articles are either focused only on adolescent psychiatric hospitals (Hutton, 2005; Gubbels et al., 2016) or staff members working in these hospitals (Duque et al., 2020; Hallman et al., 2014). To our knowledge, there are no articles focused on the impact of the built environment on both staff and patients. To bridge this gap, the authors of this study analyzed existing scholarly articles and conducted interviews at three different state hospitals with adolescent patient units. Participants’ responses to interviewer prompts highlighted specific architectural categories that can be applied to designing adolescent psychiatric hospitals.

## Method

This study investigates the influence of the built environment on the health and well-being of adolescent patients and staff of adolescent psychiatric hospitals. The first author responded to a request for proposal from one of the hospitals for which she received funding. All procedures were approved by an institutional review board (IRB). The site selection began by a web search to identify state psychiatric hospitals that have an adolescent program. The first author then emailed an invitation to the superintendent of eight hospitals and asked for their participation. Five hospitals responded, three of which got approval from their review boards to participate in the study. These hospitals (Hospitals A, B, and C) are in urban setting of different states.

### Data Collection

The data collection for this article was conducted through two phases of (1) literature analyzing and (2) interviews. The research team searched for articles published between 2000 and 2020 in the databases of PubMed, PsycINFO, ScienceDirect, Wiley Online Library, and Cambridge Core. The key words included architecture of psychiatric hospitals, adolescent psychiatric hospitals, the built environment, architecture, interior design, environmental design, and hospital design. The article elimination process was first through the title, followed by the relevancy of the abstract, and finalized by the review of the full manuscript. The result was 62 articles of which 22 were analyzed in-depth. Studies were included if they were (1) peer-reviewed and published in English, (2) reported design aspects of the hospital environment, and (3) explored the impact of the built environment on patients or staff. After potential studies were selected, the authors read and analyzed the articles individually to create a list of identified design qualities.

Interviews included questions about the participants’ use of the built environment; their needs from different sections of the hospital; and the impact of the environment on well-being, safety, and productivity of patients and staff. After the site selection and IRB approval from the hospital, the first author emailed each hospital’s superintendent and used snowballing techniques to identify and contact other participants. This technique is commonly used in qualitative research and requires a key participant to identify and contact other participants (Daly, 2007). The interviewees of this study include administrators, adolescent psychologist and psychiatrist, nurses, therapists, and peer support personnel. Sixty-nine individuals were interviewed as part of the larger study. For this analysis, only individuals who had daily interaction with adolescent patients were included in analysis (*n* = 30). Each interview lasted between 30 and 45 min. Adolescent patients were not interviewed for this study. The interviews collected qualitative data focused on attitudes toward the existing environment, perception of safety and support, patient interaction and recovery plans, and staff’s need for rejuvenation and recovery.

### Data Analysis

To establish and ensure the validity of study findings, the authors triangulated multiple data sources of literature and interviews that led to the development of a comprehensive understanding of the findings. The analysis of the literature was based on the narrative synthesis method (Popay et al., 2006) with a preliminary synthesis done individually by each author to identify and create a list of design qualities. The results were then combined, checked, modified, and verified to achieve 100% agreement by all authors. Decisions of which themes should be included were based on this study’s research question.

Qualitative interviews were recorded using an iPad and transcribed verbatim by the authors into a word document. After the first author read and de-identified the transcriptions, each author analyzed the transcriptions using a grounded theory approach that included line-by-line coding, axial coding, and comparative theoretical coding (Glaser, 1992). The authors first assigned a code to each relevant line of the transcriptions to identify ideas, thoughts, feelings, and issues mentioned by respondents (Charmaz, 2008). The codes were then sorted and compared across the responses to draw connections between created codes. This axial coding formulated categories of the codes that are most responsive to this study’s research question and trimmed away excess codes. The next phase was comparative theoretical coding (Halberg, 2006) among the sets of data collected from the three different hospitals. This process led to systematic connection and contextual arrangement of the categories. The results were initially coded and categorized by each author and then combined, checked, modified, and verified to achieve 100% agreement by all authors.

## Results and Discussion

The findings of literature analysis triangulated and juxtaposed with the results of the interview analysis informed a set of environmental design conditions that captures the complexity and connectedness of architectural design and the occupants of an adolescent psychiatric hospital. Colligating the data revealed a relationship between the built environment and the success of an adolescent psychiatric hospital. Findings suggest adolescent psychiatric hospitals must offer safe and welcoming environments that support patients’ healing and recovery as well as staff’s satisfaction. In this study, the environmental conditions of the hospital are divided to specific needs of patients and staff. Table 1 presents all categories that emerged from the data, how each category is defined in this study for adolescent patients and staff, and several subcategories that were developed through constant comparative techniques.

***The findings of literature analysis triangulated and juxtaposed with the results of the interview analysis informed a set of environmental design conditions that captures the complexity and connectedness of architectural design and the occupants of an adolescent psychiatric hospital. Colligating the data revealed a relationship between the built environment and the success of an adolescent psychiatric hospital***

**Table 1.** Environmental Design Conditions.

**Design Condition**	**Description (Environmental Qualities)**	**Subcategories (Specific Design Strategies)**
Architectural composition	*Adolescent Patients*: A built environment that offers patients freedom of movement between spaces of different sizes and affords different types of interactions *Staff*: A built environment that promotes spatial efficiency for emergency responses; a satisfactory workplace that offers social connection or separation to increase productivity	Accessibility (campus layout that avoids long distances to any patient services) Adjacency (visual and auditory access to various parts of the unit—avoid blind spots) Efficiency (campus and interior spatial layout that emphasize workflow circulation) Interior layout (physical features arranged to support various aspects of social interaction—avoid overcrowding) Versatile (multipurpose spaces that can be used for different activities and easily changed by a single user; Nanda et al., 2019) Wayfinding (physical indicators and spatial layout that offer ease of movement and navigation)
Atmosphere	*Adolescent Patients*: A home-like rehabilitating environment that provides patients with multiple spaces for different sensorial experiences and the choice of which space to be in and for how long *Staff*: A stress-free, aesthetically pleasing work environment that enhances staff well-being, satisfaction, and performance	Acoustics (architectural features that transfer pleasant sounds and prevents negative noise for patients, as well as provides quiet spaces for staff’s productivity) Autonomy (respect patients’ privacy; offer both individual and shared bedrooms) Perception (consider subjectivity and the influence of color and material on patients’ state of mind and behavior) Respite (private break areas that affords staff stress reduction)
Lighting	*Adolescent Patients*: A built environment that integrates both artificial and natural lighting to improve physical and emotional well-being which results in an accelerated recovery and regulated sleep cycle *Staff*: A built environment that integrates both artificial and natural lighting to minimize anxiety and stress, as well as increase workplace performance	Artificial lighting (noninstitutional lighting enhances safety, supports functional tasks, and provides a pleasant work environment) Natural lighting (direct and indirect natural lighting impacts circadian rhythm, decreases aggression, supports less work stress, better health status, and higher satisfaction; Shepley et al., 2017; Ulrich et al., 2018)
Natural environment	*Adolescent Patients*: A built environment with visual and physical access to plants, trees, and animals *Staff*: Exclusive nature-centric spaces for different types of activities dedicated to staff	Biophilia (architectural design to include biophilic design elements such as plants, natural material, and art for patients and staff) Courtyard and gardens (outdoor spaces for recreational activities, gardening, and play)
Safety and security	*Adolescent Patients*: A safe healing environment that discourages social conflict and prevents self-harm *Staff*: A safe working environment to reduce physical assault against staff	Boundary (physical and social restriction) High risk (provide anti-ligature equipment, safe water, and sunlight access) Visibility (reduce blind spots and maximize staff’s line of sight to spaces used by patients)

### Architectural Composition

The architectural composition of an adolescent psychiatric hospital should respond to adolescents’ psychological needs and healing process to prepare them for life outside the hospital. It is important to design the hospital in a way that patients can freely and safely move between spaces, participate in different types of therapeutic activities, and interact with each other and staff (Jovanović et al., 2019; Liddicoat, 2019). One of the participants of this study told the interviewer that “it is important to offer adolescents control over their environment and activities […] to builds up their self-esteem and help them make friends.” Some of the major concerns for adolescents with mental illness are academic difficulties, low educational achievement, low employment, social exclusion, and possible homelessness (Preyde et al., 2018). The director of patients’ rights described an ideal adolescent psychiatric hospital as a small town where buildings surround a courtyard and there is a residential unit, a school, and a therapy mall that includes various therapy rooms, a movie theater, a pharmacy, a gift store, and a restaurant to resemble a typical community outside of the hospital. This enclosed and city-like campus setting provides a serene, secure, and structured environment where adolescents can move on the path of recovery and benefit from the residential setting, academic programming, and recreational activities. These components contribute to the “overall success of the patients’ diagnostic process, treatment plan, and the adolescents’ life outside of the hospital,” said one of the psychologists interviewed for this study.

***The architectural composition of an adolescent psychiatric hospital should respond to adolescents’ psychological needs and healing process to prepare them for life outside the hospital***

#### Accessibility

The interview results presented that adolescents’ access to different services is one of the main concerns in state hospitals. These hospitals generally serve both adolescents and adults on the same campus but are required to keep the two populations completely separated for safety and security reasons. Therefore, adolescent units are built in a separate building and often far away from the adults’ units and the treatment mall, where the medical and therapeutic services are centralized. Funding limitations often lead to building one treatment mall to be shared by both populations but placed closer to adult units due to the higher number of patients. A social worker from Hospital A said that “it’s difficult to bring the children [to the treatment mall] from all the way on the other end of the hospital campus,” and the music therapist from the same hospital suggested that adolescents “need to have their own therapy spaces, a music room, art room, physical activities and a gym so they can stay active.” The adolescent unit at Hospital B was close to the rest of the campus and separated only by a courtyard, allowing patients to share all campus resources by using them at different times. The director of environmental services from this hospital said, “the kids have their own playground, but all the sports are shared and that works when we have enough staff.” While staffing is a challenge that designers do not have control over, they can address campus layout and access to a treatment mall. One solution is to build a smaller treatment mall with specific programs only for adolescent patients, while keeping some of the larger activities such as a basketball court in the main treatment mall for everyone to share.

***build a smaller treatment mall with specific programs only for adolescent patients, while keeping some of the larger activities such as a basketball court in the main treatment mall for everyone to share.***

#### Adjacency

Participants expressed the need for staff’s visual and audible access to patients from the nurse station as a necessity of the hospital design. Adjacency of communal spaces with the nurse stations offers patients an increased feeling of freedom and security (Jovanović et al., 2019) which can help adolescents develop independence, provides a safe space for them to gather around (Shepley et al., 2016) to increase opportunities for positive interaction, and have a higher level of connection with staff (Shattell et al., 2008). This also allows staff to monitor patients more closely and offer “comfortable seating for quiet activities near the nurse stations,” said a nurse at Hospital A. While staff have varying opinions on open or closed nurse stations, “patients mostly prefer open without any barrier such as a plexiglass panel,” said one of the activity nurses at Hospital C. A nurse manager of the adolescent unit at Hospital B stated “instead of a glass bubble, we have an open desk in front where patients gather to talk, read, or work on a puzzle. The nurses’ offices are in the back of the hub.”

#### Efficiency

To better serve patients, staff need clear and quick access to different sections of the adolescent unit as well as different buildings of the hospital. Circulation and campus planning impact both functional and psychological aspects of the workflow by providing fundamental support to ease the hardship of everyday routines (Jiang, & Verderber, 2016). It is important to consider the fastest and safest path for staff to move between essential locations for patient care and self-care. Multiple staff members in Hospital A voiced their concerns about unnecessary distance and wasted time between the adolescent unit and other units of the hospital when they need to respond to emergencies.

#### Interior layout

It is important to recognize the appropriate spatial layout and physical spaces of an adolescent psychiatric hospital in a way that serves both patients and staff. An open floor plan is more preferred as it increases face-to-face communication, team interaction, and interdisciplinary collaboration (Jovanović et al., 2019; Liddicoat, 2019). The nurse manager from Hospital C said, “an open floor plan gives adolescents the freedom of moving in different spaces while monitored by staff without being told they can or cannot go somewhere. It is just easier for everyone.” Adolescents often have difficulties with interpersonal relationships, social situations, managing psychiatric symptoms, stigma, and bullying (Preyde et al., 2018). A physician at Hospital A talked about the importance of replicating family life and social activity in the community to encourage positive interactions with peers and improve adolescent patients’ sense of self-worth. An adolescent psychologist from Hospital C emphasized on the importance of active and passive behaviors for adolescent patients and said “there needs to be a place where they can run, be loud, be teenagers. But they also need a place to practice being calm.” Examples of interior spaces that promote active interaction include exercise and play areas for patients to get engaged in positive movement and exert physical energy (Trzpuc et al., 2016) and “supervised woodshop to express creativity with hands-on craft” said the director of vocational services in Hospital A. Spaces that inspire passive interactions include a library, a greenhouse, indoor plants, a spiritual room (interviewees), quiet rooms, sensory rooms, dayrooms, and patient rooms with front porches (Shepley et al., 2016). Offering different types of spaces provide adolescents a variety of privacy and opportunities to come together, socialize, and bond (Hutton, 2005).

#### Versatility

Versatility is defined as a multipurpose space that includes built-ins with multiple uses and can be changed by a single user within minutes (Nanda et al., 2019). Flexible spaces provide options for spatial configuration and cater to a wide range of social activities for adolescent patients. Participants of this study asked for flexible interior spaces that can offer different activities such as “reading, relaxing, playing games, and watching TV” to help adolescents avoid boredom and afford them control over the type of activity they want to participate in. The Performance Program Supervisor of Hospital B expressed the need for larger rooms that can be easily divided and used for different therapy sessions at the same time. The music therapist of Hospital A said “musical instruments are expensive, and we cannot afford buying multiple of the same instrument for different units. So, we need a versatile space that allows the equipment in the same place to be used at different times.” Therefore, it is important to design versatile spaces with socio-petal furniture arrangements to promote positive social interaction, decrease isolation (Jovanović, 2019), facilitate personal space regulation, and provide occupants control of their environment (Shepley et al., 2016; Ulrich et al., 2018) in adolescent psychiatric hospitals.

#### Wayfinding

The ability for adolescent psychiatric patients to successfully navigate through a care center is crucial for their daily routine activities. Wayfinding should be recognized as a high priority when designing psychiatric hospitals as it can be a challenge in large hospital campuses with several buildings that lack distinctive appearance (Devlin, 2014). Inadequate wayfinding and signage can cause aggression or abusive behavior among patients (Liddicoat, 2019). The adolescent psychologist from Hospital A emphasized the importance of wayfinding by saying “these are kids, and they have less patience than adults […] they need to go where they want and get what they want as quickly as possible.” A wayfinding system needs to be a navigable and readable environment (Shepley et al., 2017) and avoid “long corridors, repetitive elements, and changes of direction within the circulation system” (Devlin, 2014, pp. 425–426).

### Atmosphere

Atmosphere is defined as an emotional reaction to a specific space and therefore cannot be directly constructed but emerges from the built environment and its impact on the occupants (Norouzi, 2016). Different architectural characteristics create different atmospheres (Norouzi et al., 2019). Examples of these characteristics are noise, color, material, texture, and form that influence spatial intimacy (Zumthor, 2006), privacy, and autonomy (Norouzi et al., 2019). It is important to consider the atmosphere of an adolescent psychiatric hospital as it enhances staff and patients’ satisfaction (Jovanović et al., 2019) and well-being (Sheehan et al., 2013), increases social engagement (Trzpuc at al., 2016), and reduces stress (Ulrich et al., 2018).

#### Acoustics

Sound and noise can range from stressful to soothing and therapeutic and impact staff, patients, and visitors of a hospital. An appropriate acoustic environment benefits staff’s well-being and patients’ therapy process (Zhou et al., 2020). However, appropriate acoustic design has not been a priority in psychiatric hospitals (Shepley et al., 2017). Unwanted sounds and noise such as traffic, trains, airports, and community noise within a hospital unit can hinder staff’s work productivity and communication quality with patients while causing psychological distress and helplessness among patients (Shepley et al., 2017; Ulrich et al., 2018). The director of admissions in Hospital B said, “we need a quiet therapeutic space as staff break room in each unit so they can relax and unwind but available to respond to emergencies when needed.” A physical therapist from Hospital C said, “the breeze through the trees, birds chirping, and sound of water create a calm environment for patients and staff. […], there needs to be a way to have pleasant sound both inside and outside the unit.” Incorporating acoustic floor and ceiling tiles, wall panels, movable partition panels, and solid core doors can mitigate unwanted noise transmissions (Liddicoat, 2019). Providing single bedrooms in adolescent psychiatric hospitals enhances privacy and reduces noise levels from adjacent patient neighbors (Ulrich et al., 2018). Indoor sensory rooms can provide opportunities for patients to listen to music or pleasant nature sounds.

#### Autonomy

Autonomy, as independence or freedom of choice, is an element often forgotten in psychiatric hospitals and even more so for adolescents. Respecting patients’ autonomy starts by providing privacy options. Lack of privacy results in heightened anxiety levels, stress, and aggression (Liddicoat, 2019). A way to provide privacy is through offering single bedrooms for patients. Single bedrooms with private bathrooms encourage social engagement (Trzpuc et al., 2016); offers therapeutic security and comfort, expression of ownership, opportunity to personalize and decorate the space (Shepley et al., 2017), and freedom to withdraw when feeling unwell (Jovanović et al., 2019); and improve the dignity and safety of patients (Sheehan et al., 2013). Private bedrooms can provide a greater “sense of personal control and security,” said one of the interviewees. This can be provided by offering access to technology such as television for entertainment, phone, or computer to stay connected with family, control over the temperature of their room for comfort, private lockers to store their belongings in their bedroom, and most importantly options in the type of activities to be involved in.

#### Perception

Offering choices increases an individual’s sense of control and can provide opportunities for patients to modify and personalize their environment based on personal perceptions (Bergamin et al., 2022; Pasha & Shepley, 2017). While this is important and possible for personal spaces, perception is based on interpretation of experiences (Pasha & Shepley, 2017) and varies for different people. Therefore, it is difficult to accommodate in designing public spaces. One of the most recognized topics in relation to psychiatric hospitals is creating a deinstitutionalized and homelike environment. Previous studies presented homelike environments can be created through light and soothing colors, cheerful and welcoming facade and entrances, use of local materials and design characteristics (Li, 2018), clean floors and comfortable furniture (Shepley et al., 2017), nontextured interior walls (Wang et al., 2020), view of nature and landscape painting (Evans, 2003), and indoor plants (Duque et al., 2020) to improve patients and staff’s satisfaction. Although these points are agreed upon in most research studies, the perception of the space is influenced by individuals’ associational meaning of the surrounding environment (Wang et al., 2020) and can be different for many individuals. Participants described a homelike environment as a comfortable place with “bright colors, familiar items from home, a way to listen to music and podcasts, natural light, comfortable flooring, nice variety of indoor plants, and outdoor gardens.” However, the concept of comfort was not defined by any of the participants.

Another controversial subject related to perception is color. A music therapist interviewed for this study, said “bright colors could be very over stimulating because some folks have sensory deficits.” Yildirim et al. (2011) confirms this by stating that warm colors provoke active feelings and, in some cases, increase anxiety. Contrary to these statements, the chief of psychiatry in Hospital B suggested that “warmer colors are better than cool blues for the common areas because they encourage interaction and communication.” One staff member interviewed for this study said, “anything but light blues and greens […] that feels like an asylum from the old movies; very institutionalized, not homey at all,” but Yildirim et al. (2011) suggest that cool colors make the space feel peaceful, relaxing, and calm.

#### Respite

In response to a highly stressful work environment of a psychiatric hospital and to contribute to the staff’s reduction of stress, burnout, and fatigue, while offering them opportunities to relax, focus, and concentrate (Nejati et al., 2015), it is important to create indoor and outdoor relaxation and rejuvenation areas specific to staff. Interviewees of this study asked for a balance between passive and active respite such as a meditation room and a basketball court. Areas of respite serve as a place to withdraw and seek refuge from patients and families along with allowing privacy and socialization with coworkers (Liddicoat, 2019). The staff break area should include artwork, indoor plants, windows with outdoor views and access to nature by offering opportunities to walk through gardens, receive direct sunlight, and listen to the sound of birds or water (Nejati et al., 2015). Interviewees of this study described the importance of incorporating access to covered patios, fitness areas, and outdoor walking trails and asked for centralized break rooms near work areas, so they can step away from a hectic work environment, think and regroup their thoughts.

***it is important to create indoor and outdoor relaxation and rejuvenation areas specific to staff***

### Lighting

Exposure to light impacts the circadian rhythm which synchronizes the body’s internal clock and regulates the sleep cycle (Joseph, 2006). Inadequate exposure to light can lead to reduced cognitive abilities, anxiety, drowsiness, fatigue, stress, and depression, along with vulnerability to seasonal affective disorder (Evans, 2003; White et al., 2013). Therefore, integrating natural and artificial light in the design of an adolescent psychiatric hospital is essential for improving workplace performance for staff and enhancing patients’ recovery process. It is necessary to incorporate windows, and skylights allow natural light into the built environment (Jiang & Verderber, 2016) and select appropriate noninstitutional lighting fixtures to provide adequate light distribution for day and night usage.

#### Artificial lighting

Lighting is linked with a sense of comfort and well-being. Consequently, when daylight is not sufficient, artificial lighting must compensate for and provide adequate lighting that extends the field of view in play areas for adolescent patients and creates ideal working conditions for staff. Electric lighting is used to positively impact mood, circadian rhythm, sleep cycle, and task performance and decrease depression and length of stay in a hospital (Shepley et al., 2017). Adequate exposure to artificial light is essential for night-shift staff in a psychiatric hospital as it readjusts their circadian rhythm and helps with irregular sleep–wake schedules that could lead to health problems (Joseph, 2006). Artificial lighting design considerations include glare and shadow-free light distribution, daylight sensors, dimmers, switch controllers, and high and uniform luminance.

#### Natural lighting

Daylight should be prioritized when designing an adolescent psychiatric hospital as it affects staff and patients’ physical and psychological health. Inadequate light has a direct effect on fatigue, insomnia, and other psychiatric diseases (Evans, 2003; Nabil & Mardaljevic, 2006; Shepley et al., 2016). Exposure to natural lighting can impact staff and patients by enabling performance of visual tasks (Peek-Asa et al., 2009), affecting mood and perception (Partonen & Lonnqvist, 2000), controlling the body’s circadian system and improving sleep cycle (Boubekri et al., 2014), decreasing aggression (Ulrich et al., 2014), depression, and anxiety as well as facilitating wayfinding (Evans, 2003; Liddicoat, 2019; Shepley et al., 2017). The music therapist in Hospital A mentioned the importance of integrating natural daylight in adolescent psychiatric hospitals and said, “the kids are generally happier and more willing to sit through the therapy session in the room with windows and sunlight.”

### Natural Environment

Connection and access to the natural environment can positively impact physical and mental health of individuals by improving cognitive abilities, increasing the sense of belonging and self-worth (Hammell, 2021), helping patients focus on their inner healing resources, and offering staff a place of respite and visitors a relaxed setting to interact with loved ones (Marcus, 2007). Designing a connection to the natural environment in an adolescent psychiatric hospital can be accomplished through biophilic design with the goal of providing a healing environment that promotes well-being for staff and patients. This could be achieved by offering direct and indirect connections to natural elements and incorporating nature-centric spaces with activities ranging from passive to active (Marcus, 2007). Interviewees described nature-centric environments as spaces that include plants, trees, natural materials, artwork, color, water features, and animals. Interviewees also mentioned Zen gardens, planters, green courtyards, and walking trails as examples of places that provide opportunities for different levels of activities.

#### Biophilia

Biophilic design focuses on incorporating nature into the built environment to advance people’s health, fitness, and well-being. Integrating biophilia in the design of an adolescent psychiatric facility can promote passive and active interactions. Marcus (2007) lists these interactions as viewing the garden through a window, sitting outside, dozing/napping/meditating, gentle rehabilitation exercises, walking to preferred spots, raised bed gardening, and participating in sports. Designing passive connections to nature can create a temporary escape from an oppressive atmosphere, offering hope of rapid recovery, along with experiencing the change in time, throughout the day and improving the quality of daily routines for staff and patients (Nejati et al., 2015). An intervention coach from Hospital C said, “more open spaces with outdoor visibility and decorative paneling or paintings of nature in different spaces will help patients feel at peace.” Nature art presents distinct synergistic benefits that can impact the psychosocial well-being of patients by increasing self-esteem and decreasing social isolation (Thomson et al., 2020). Displaying nature art can reduce patient anxiety and agitation (Shepley et al., 2017) and decrease the use of anxiety medication (Nanda et al., 2019). The dentist in Hospital A said, “there’s a mural in our main walkway that has trees, koala bears, giraffes, and many patients love it” and “it makes them happy; it makes them think of something besides their delusions. It’s relaxing.” An adolescent patient who had learned about architects interviewing members of the hospital wrote a message and asked a nurse to deliver it to the research team. The note read,

I like to see artwork that is made by some of us. There are people here, like this guy in my unit who draws very nicely. We can have his drawings on the wall. The big sculptures in the garden are nice too; specially to look at and relax on a sunny day.

#### Courtyard

Courtyards, outdoor spaces surrounded by buildings, can provide opportunities for recreational activities, play, and gardening. Interviewees of this study requested having multiple small courtyards accessible to patients for different types of activities. It is important for adolescent patients to have a place to exert their energy throughout the day (Shepley et al., 2016), and they are most likely to be physically active in an outdoor environment that includes greenery (Gubbels et al., 2016). Patients with the opportunity to walk in a garden, be around diverse plants and flowers, listen to the sound of water, and receive direct sunlight are reported to be less stressed (Nejati et al., 2015). The interviewees of this study expressed interests for walking trails that are close to buildings where both patients and staff can go on daily walks. They also asked for outdoor areas that are set up for sports, relaxation, weight training, play structure, running, and biking, and outdoor shaded seating areas for staff.

Although there were inquiries for different types of gardens, the most requested green spaces were therapeutic and vegetable gardens. Most interviewees indicated that attributes of a healing environment as spending time with plants and water features. These activities can reduce anxiety, provide positive distractions for patients, and offer them opportunities to be involved in their own recovery and healing process (Liddicoat, 2019). This can also allow staff to cope with bereavement, process stressful work situations, spend quality time, and work with adolescent patients outside of the hospital building (Marcus, 2007). Therapeutic gardens should provide views to the sky and changing cloud formations, a plentiful supply of plant materials with distinctive seasonal changes, subtleties of color and texture, with leaves or grass that move with the slightest breeze (Marcus, 2007), as well as safe water features where patients can see and hear the water but not drink it. Interviewees talked about the benefits of gardening and planting vegetables to be tremendous for patients as it could help them practice acceptance, understanding the process of growth, living in the moment, and eating healthy.

### Safety and Security

Safety is a major factor in adolescent psychiatric hospitals, as patients can become violent and aggressive when irritated. Tense or escalating situations often arise between patients in psychiatric hospitals in which staff members need to intervene. Design features such as efficient visibility and circulation that maximizes line of sight throughout the facility, reduces blind spots from corridor nooks, and provides multiple exit points that improve accessibility, as well as anti-ligature equipment, durable and heavy furniture, and shatterproof windows (Carr, 2017).

#### Boundary

Physical indicators that define the use of space can allow for different types and levels of interaction by determining separations or offering connections (Norouzi et al., 2019). A nurse in Hospital C said, “boundaries are for connection as much as they are for separation […], windows connect the patients to the outdoors even on a hot day when they can’t go outside.” The nurse continued by saying: Windows from the patients’ room to the hallway offer safety and privacy by allowing staff to check on patients without entering the room. Boundaries for separation are essential for safety and security to keep adolescents in their own healing environment and away from adult patients and the public.

#### High risk

Equipment that can be used by patients for self-harm are considered high-risk factors. These can be eliminated with the appropriate use of durable building materials, damage-resistant furniture, and anti-ligature equipment (Carr, 2017; Shepley, 2017). The head nurse in Hospital A described the importance of avoiding sharp corners and objects used throughout the unit in which patients can use to harm themselves or others.

#### Visibility

Patient visibility and efficient circulation are imperative when designing an adolescent psychiatric hospital, as it has a direct influence on the safety of staff and patients. A central nurse station within an adolescent unit is crucial for providing staff with maximum patient visibility (Shepley et al., 2016). Although open nurse stations provide better visibility and support staff capability to anticipate and prevent aggressive behavior (Ulrich et al., 2018), most staff members at Hospitals A and C said that they feel safer in enclosed stations. In other studies, decentralized nurse stations positively influenced proximity to patients in a larger design footprint as well as time spent with patients (Fay et al., 2017). Another important factor to consider are blind spots that can be created by walls or furniture. Interviewees suggested the use of convex mirrors or cameras to offer visibility. Although video surveillance provides additional security and can increase patients’ choice about monitoring options, it can have adverse effects on patients’ well-being and cause symptoms of fear, distrust, and paranoia (Appenzeller et al., 2019). These dubious facts are due to lack of imperial evidence and lead to difficulty in considering video surveillance beneficial or hindering. We recommend that future studies to consider representing adolescents in the design of psychiatric hospitals by including them in the interviews or surveys for data collection.

## Conclusion

The design of an adolescent psychiatric hospital from identifying the client needs to architectural programming and design must be researched based. This study has evaluated the impact of the built environment on staff and patients in an adolescent psychiatric hospital and identified a set of important design conditions of architectural composition, atmosphere, lighting, natural environment, safety, and security. Incorporating these conditions into architectural design of adolescent psychiatric hospitals will benefit staff, patients, and their family members. The specific design strategies that need to be incorporated in the architectural design of a safe and secure adolescent psychiatric hospital include an open floor plan that respects patients’ autonomy and offers privacy while always providing staff with full visibility of patients. Adolescents’ needs from the built environment is connected to their social and developmental needs of control, opportunities for individual growth, and positive peer-socialization. A campus with a residential unit, a school, and a therapy mall where buildings surround courtyards and play spaces provides a safe and secure environment for adolescent patients’ self and academic growth as well as socialization and friendship. These design elements can also be implemented in the renovation of existing adolescent psychiatric hospitals.

## Implications of Practice

- Architectural composition: When configuring the layout of an adolescent psychiatric hospital, flexibility is important to promote efficiency for staff and social interactions with patients by taking into consideration accessibility, interior layout, versatility, wayfinding, and adjacency.
- Atmosphere: The atmosphere of a space prioritizes a peaceful, home-like environment for patients and staff which enhances autonomy, perception, respite, and pleasant acoustics.
- Lighting: Taking into consideration, natural and artificial lighting is vital in promoting an accelerated recovery process for patients while also increasing staff productivity.
- Natural environment: Introducing elements of the natural environment that encourage passive or active interactions among patients and staff can create a therapeutic healing environment furthering well-being.
- Safety and security: It is necessary to provide a safe environment that maximizes staff visibility toward patients, utilization of anti-ligature equipment for mitigating self-harm, and integrates appropriate boundaries throughout an adolescent psychiatric hospital.

## References

[bibr1-19375867231180907] Appenzeller E. Appelbaum P. S. Trachsel M. (2020). Ethical and practical issues in video surveillance of psychiatric units. Psychiatric Services (Washington, D.C.), 71(5), 480–486. 10.1176/appi.ps.201900397 31847737

[bibr2-19375867231180907] Bergamin, Luigjes J. Kiverstein J. Bockting C. L. Denys D . (2022). Defining autonomy in psychiatry. Frontiers in Psychiatry, 13, 801415–801415. 10.3389/fpsyt.2022.801415 35711601 PMC9197585

[bibr3-19375867231180907] Blumberg R. Devlin A. S. (2006). Design Issues in hospitals: The adolescent client. Environment and Behavior, 38(3), 293–317. 10.1177/0013916505281575

[bibr4-19375867231180907] Borg-Laufs M. (2013). Basic psychological needs in childhood and adolescence. Journal of Education and Research, 3, 41–51. 10.3126/jer.v3i0.7851

[bibr5-19375867231180907] Boubekri M. Cheung I. N. Reid K. J. Kuo N. W. Wang C. H. Zee P. C . (2014). Impact of windows and daylight exposure on overall health and sleep quality of office workers-A case-control pilot study. Journal of Clinical Sleep Medicine, 10(6), 603–611. 24932139 10.5664/jcsm.3780 PMC4031400

[bibr6-19375867231180907] Carr R. F . (2017). Psychiatric facility. WBDG Health Care Subcommittee. https://www.wbdg.org/building-types/health-care-facilities/psychiatric-facility

[bibr7-19375867231180907] Charmaz K. (2008). Constructionism and the grounded theory method. In Holstein J.A. Gubrium J. F. (Eds.), Handbook of constructionist research (pp. 397–412). The Guilford Press.

[bibr8-19375867231180907] Chun T. H. Mace S. E. Katz E. R. , American Academy Of Pediatrics, Committee On Pediatric Emergency Medicine, A. A. C. O. E. P., & Pediatric Emergency Medicine Committee. (2016). Evaluation and management of children and adolescents with acute mental health or behavioral problems. Part I: Common clinical challenges of patients with mental health and/or behavioral emergencies. https://pubmed.ncbi.nlm.nih.gov/27550977/ 10.1542/peds.2016-1570 27550977

[bibr9-19375867231180907] Daly K . (2007). Qualitative methods for family studies and human development. Sage.

[bibr10-19375867231180907] Devlin A. S. (2014). Wayfinding in healthcare facilities: Contributions from environmental psychology. Behavioral Sciences, 4(4), 423–426. 10.3390/bs4040423 25431446 PMC4287692

[bibr11-19375867231180907] Duque M. Annemans M. Pink S. Spong L. (2020). Everyday comforting practices in psychiatric hospital environments: A design anthropology approach. Journal of Psychiatric and Mental Health Nursing, 1–12. 10.1111/jpm.12711 33185312

[bibr12-19375867231180907] Evans G. (2003). The built environment and mental health. Journal of Urban Health: Bulletin of the New York Academy of Medicine, 80, 536–555. 14709704 10.1093/jurban/jtg063 PMC3456225

[bibr13-19375867231180907] Fay L. Carll-White A. Schadler A. Isaacs K. B. Real K. (2017). Shifting landscapes: The impact of centralized and decentralized nursing station models on the efficiency of care. Health Environments Research & Design Journal, 10(5), 80–94. 10.1177/1937586717698812 28359162

[bibr14-19375867231180907] Glaser B. (1992). Emergence vs. forcing: Basics of grounded theory analysis. Sociology Press.

[bibr15-19375867231180907] Gubbels J. Kremers S. Droomers M. Hoefnagels C. Stronks K. Hosman C. de Vries S. (2016). The impact of greenery on physical activity and mental health of adolescent and adult residents of deprived neighborhoods: A longitudinal study. Health & Place, 40, 153–160. 10.1016/j.healthplace.2016.06.002 27322564

[bibr16-19375867231180907] Hallberg L. (2006). The “core category” of grounded theory: Making constant comparisons. International Journal of Qualitative Studies on Health and Well-Being, 1(3), 141–148. 10.1080/17482620600858399

[bibr17-19375867231180907] Hallman I. O’Connor N. Hasenau S. Brady S. (2014). Improving the culture of safety on a high-acuity inpatient child/adolescent psychiatric unit by mindfulness-based stress reduction training of staff. Journal of Child and Adolescent Psychiatric Nursing, 27(4), 183–189. https://doi-org.libweb.lib.utsa.edu/10.1111/jcap.12091 25382746 10.1111/jcap.12091

[bibr18-19375867231180907] Hammell K. (2021). Occupation in natural environments; Health equity and environmental justice. Canadian Journal of Occupational Therapy (1939), 88(4), 319–328. 10.1177/00084174211040000 34486421

[bibr19-19375867231180907] Hutton A. (2005). Consumer perspectives in adolescent ward design. Journal of Clinical Nursing, 14(5), 537–545. 10.1111/j.1365-2702.2004.01106.x 15840067

[bibr20-19375867231180907] Jiang S. Verderber S. (2016). On the planning and design of hospital circulation zones: A review of the evidence-based literature. Health Environments Research & Design Journal, 10(2), 1–23. 10.1177/1937586716672041 27742819

[bibr21-19375867231180907] Jones V. (2019). Youth access to mental health services is an un-prioritized civil right. American Bar Association. Retrieved December 7, 2020, from https://www.americanbar.org/groups/litigation/committees/diversity-inclusion/articles/2019/youth-access-to-mental-health-civil-rights/

[bibr22-19375867231180907] Joseph A. (2006). Impact of light on outcomes in healthcare settings. The Center for Health Design. Retrieved February 12, 2021, from https://www.healthdesign.org/chd/research/impact-light-outcomes-healthcare-settings

[bibr23-19375867231180907] Jovanović N. Campbell J. Priebe S. (2019). How to design psychiatric facilities to foster positive social interaction—A systematic review. European Psychiatry, 60, 49–62. 10.1016/j.eurpsy.2019.04.005 31112827

[bibr24-19375867231180907] Li X . (2018). How architecture can promote a sustainable and therapeutic experience for patients in psychiatric hospitals in China [Master’s thesis, Rochester Institute of Technology]. RIT Scholar Works.

[bibr25-19375867231180907] Liddicoat S. (2019). Designing a supportive emergency department environment for people with self harm and suicidal ideation: A scoping review. Australasian Emergency Care, 22(3), 139–148. 10.1016/j.auec.2019.04.006 31129061

[bibr26-19375867231180907] Marcus C. C. , (2007). Healing gardens in hospitals. Design and Health, 1(1). Retrieved January 12, 2021, from https://intogreen.nl/wp-content/uploads/2017/07/cooper_marcus.pdf

[bibr27-19375867231180907] McGorry B. Bates T. Brichwood M. (2013). Designing youth mental health services for the 21st century: Examples from Australia, Ireland and the UK. British Journal of Psychiatry, 202(s54), s30–s35. 10.1192/bjp.bp.112.119214 23288499

[bibr28-19375867231180907] Nabil A. Mardaljevic J. (2006). Useful daylight illuminances: A replacement for daylight factors. Energy and Buildings, 38(7), 905–913. 10.1016/j.enbuild.2006.03.013

[bibr29-19375867231180907] Nanda U. Hoelting M. Fuassel W. (2019, June 14). FleXX: A study of flexibility in outpatient settings. Retrieved December 30, 2020, from https://www.hksinc.com/how-we-think/research/flexx-a-study-of-flexibility-in-outpatient-settings/

[bibr30-19375867231180907] Nejati A. Shepley M. Rodiek S. (2015). Restorative design features for hospital staff break areas: A multi-method study. Health Environments Research & Design Journal, 9(2). https://doi-org.libweb.lib.utsa.edu/10.1177/1937586715592632 10.1177/1937586715592632 26163571

[bibr31-19375867231180907] Norouzi N. (2016). Intergenerational facilities: Designing intergenerational space through a human development lens [Doctoral dissertation, Virginia Tech]. Vtechworks.

[bibr32-19375867231180907] Norouzi N. Jarrott S. Chaudhury H. (2019). Designing intergenerational space through a human-development lens. Journal of Architectural and Planning Research, 36(1), 35–51.

[bibr33-19375867231180907] Partonen T. Lonnqvist J. (2000). Bright light improves vitality and alleviates € distress in healthy people. Journal of Affective Disorders, 57(1), 55–61. 10708816 10.1016/s0165-0327(99)00063-4

[bibr34-19375867231180907] Pasha S. Shepley M. (2017). Design for mental and behavioral health. Taylor & Francis. 10.4324/9781315646916

[bibr35-19375867231180907] Peek-Asa C. Casteel C. Allareddy V. Nocera M. Goldmacher S. OHagan E. Blando J. Valiante D. Gillen M. Harrison R . (2009). Workplace violence prevention programs in psychiatric units and facilities. Archives of Psychiatric Nursing, 23(2), 166–176. 19327559 10.1016/j.apnu.2008.05.008

[bibr36-19375867231180907] Popay J. Roberts H. Sowden A. Arai L. Rodgers M. Britten N. Roen K. Duffy S. (2006). Guidance on the conduct of narrative synthesis in systematic reviews. Retrieved December 28, 2020, from https://citeseerx.ist.psu.edu/viewdoc/download?doi=10.1.1.178.3100&rep=rep1&type

[bibr37-19375867231180907] Preyde M. Parekh S. Heintzman J. (2018). Youths’ experiences of school re-integration following psychiatric hospitalization. Journal of the Canadian Academy of Child and Adolescent Psychiatry, 27(1), 22–32. 29375630 PMC5777688

[bibr38-19375867231180907] Shattell M. Andes M. Thomas S. (2008). How patients and nurses experience the acute care psychiatric environment. Nursing Inquiry, 15(3), 242–250. 10.1111/j.1440-1800.2008.00397.x 18786217

[bibr39-19375867231180907] Sheehan B. Burton E. Wood S. Stride C. Henderson E. Wearn E. (2013). Evaluating the built environment in inpatient psychiatric wards. Psychiatric Services, 64(8), 789–795. 10.1176/appi.ps.201200208 23632426

[bibr40-19375867231180907] Shepley M. Pasha S . (2017). Design for mental and behavioral health (1st ed.). Routledge. 10.4324/9781315646916

[bibr41-19375867231180907] Shepley M. Watson A. Pitts F. Garrity A. Spelman E. Fronsman A. Kelkar J. (2016). Mental and behavioral health environments: critical considerations for facility design. General Hospital Psychiatry, 42, 15–21. 10.1016/j.genhosppsych.2016.06.003 27638966

[bibr42-19375867231180907] Shepley M. Watson A. Pitts F. Garrity A. Spelman E. Fronsman A. Kelkar J. (2017). Mental and behavioral health settings: Importance & effectiveness of environmental qualities & features as perceived by staff. Journal of Environmental Psychology, 50, 37–50. 10.1016/j.jenvp.2017.01.005

[bibr44-19375867231180907] Thomson L. Morse N. Elsden E. Chatterjee H. (2020). Art, nature and mental health: assessing the biopsychosocial effects of a “creative green prescription” museum programme involving horticulture, artmaking and collections. Perspectives in Public Health, 140(5), 277–285. 10.1177/1757913920910443 32449492 PMC7498904

[bibr45-19375867231180907] Trzpuc S. Wendt K. Heitzman S. Skemp S. Thomas D. Dahl R. (2016). Does space matter? An exploratory study for a child–adolescent mental health inpatient unit. Health Environments Research & Design Journal, 10(1), 23–44. 10.1177/1937586716634017 27081193

[bibr46-19375867231180907] Ulrich R. Bogren L. Gardiner S. Lundin S. (2018). Psychiatric ward design can reduce aggressive behavior. Journal of Environmental Psychology, 57, 53–66. 10.1016/j.jenvp.2018.05.002

[bibr47-19375867231180907] Ulrich R. Bogren L. Lundin S. (2014). Towards a design theory for reducing aggression in psychiatric facilities [Conference session]. In Arch 12: Architecture/research/care/health conference. Chalmers Institute of Technology, Gothenburg, Sweden.

[bibr48-19375867231180907] Wang C Lu W. Ohno R. Gu Z . (2020). Effect of wall texture on perceptual spaciousness of indoor space. International Journal of Environmental Research and Public Health, 17(11), 4177. 10.3390/ijerph17114177 32545379 PMC7312763

[bibr49-19375867231180907] White M. D. Ancoli-Israel S. Wilson R. R. (2013). Senior living environments: Evidence-based lighting design strategies. Health Environments Research & Design Journal, 7(1), 60–78. https://doi-org.libweb.lib.utsa.edu/10.1177/193758671300700106 24554316 10.1177/193758671300700106

[bibr50-19375867231180907] Yildirim H. Hidayetoglu M. F. Capanoglu A. (2011). Effects of interior colors on mood and preference: Comparisons of two living rooms. Perceptual and Motor Skills, 112(2), 509–524. 10.2466/24.27.PMS.112.2.509-524 21667759

[bibr51-19375867231180907] Zhou T. Wu Y. Meng Q. Kang J. (2020). Influence of the acoustic environment in hospital wards on patient physiological and psychological indices. Frontiers in Psychology, 11, 1600–1600. 10.3389/fpsyg.2020.01600 32848994 PMC7396688

[bibr52-19375867231180907] Zumthor P . (2006). Atmospheres. Birkhauser.

